# Thyroid carcinoma and primary amenorrhea due to Mayer-Rokitansky-Küster-Hauser syndrome: a case report

**DOI:** 10.1186/1752-1947-6-377

**Published:** 2012-11-06

**Authors:** Doina Piciu, Andra Piciu, Alexandru Irimie

**Affiliations:** 1 Department of Endocrine Tumors and Nuclear Medicine, Institute of Oncology, ‘Prof. Ion Chiricuţă’, 34-36 Republicii St, Cluj-Napoca, 400015, Romania; 2 University of Medicine and Pharmacy, ‘Iuliu Haţieganu’, 8 Victor Babes St, Cluj-Napoca, 400023, Romania; 3 Department of Oncology, University of Medicine and Pharmacy, ‘Iuliu Haţieganu’, 8 Victor Babes St, Cluj-Napoca, 400023, Romania

**Keywords:** Thyroid carcinoma, Mayer-Rokitansky-Küster-Hauser syndrome

## Abstract

**Introduction:**

This case report describes an association between an exceptionally rare congenital anomaly and differentiated thyroid carcinoma. Mayer-Rokitansky-Küster-Hauser syndrome is characterized by vaginal aplasia associated with other Müllerian duct abnormalities. Its penetrance varies, as does the involvement of other organs. The association with thyroid carcinoma appears to be exceptionally rare, and warrants further attention.

**Case presentation:**

We present the case of a 19-year-old Caucasian woman with amenorrhea and thyroid disease, with an unusually late diagnosis of gynecological abnormality. Management of her amenorrhea included investigation for congenital anomalies, including Mayer-Rokitansky-Küster-Hauser syndrome. Endocrine evaluation included a detailed analysis of sex hormone levels and thyroid function. The results of a physical examination and neck ultrasonography revealed abnormalities of the thyroid gland, which led to a diagnosis of thyroid cancer. To the best of our knowledge, thyroid cancer has not previously been reported in association with Mayer-Rokitansky-Küster-Hauser syndrome. However, genetic links between Mayer-Rokitansky-Küster-Hauser syndrome and thyroid cancer have not been investigated. The association may therefore be coincidental.

**Conclusions:**

All women with primary amenorrhea should undergo complete investigation of the genital tract and the endocrine axis. Careful examination of the thyroid gland is recommended.

## Introduction

Mayer-Rokitansky-Küster-Hauser (MRKH) syndrome is characterized by vaginal aplasia associated with other Müllerian duct abnormalities. The severity of the anomaly varies, as does the number of involved organs. The prevalence is one in 5000 women [1]. In type I MRKH syndrome, there is an isolated absence of the proximal two-thirds of the vagina. In type II MRKH syndrome, there are associated malformations including vertebral, cardiac, urological (upper tract), and otological anomalies [2]. The extent of the vaginal aplasia varies in both types, ranging from a virtually absent vagina to a short vagina measuring 2cm to 5cm in length (normal range 8cm to 12cm). Patients have normal development of secondary sexual characteristics, and MRKH syndrome is usually undetected until the patient is referred for investigation of primary amenorrhea. This syndrome is important because it is the second most common cause of primary amenorrhea, after gonadal pathology. The psychological consequences are severe, even if the anatomical defects can be surgically treated. Surgery allows patients to achieve normal sexual function and even assisted reproduction.

## Case presentation

A 19-year-old Caucasian woman was referred to our clinic for evaluation of amenorrhea. This was an unusually late presentation for the investigation of primary amenorrhea. She had been investigated for thyroid dysfunction as the presumed cause of her amenorrhea at the age of 14 years. Her serum thyroid hormone levels were normal at that time, with thyroid stimulating hormone (TSH) level of 1.4mIU/L (normal range 0.4 to 4.2mIU/L) and free thyroxine (FT4) level of 14.6pmol/L (normal range 12 to 2.4pmol/L), and no further investigation was undertaken. There was no family history of thyroid cancer.

**Figure 1:**
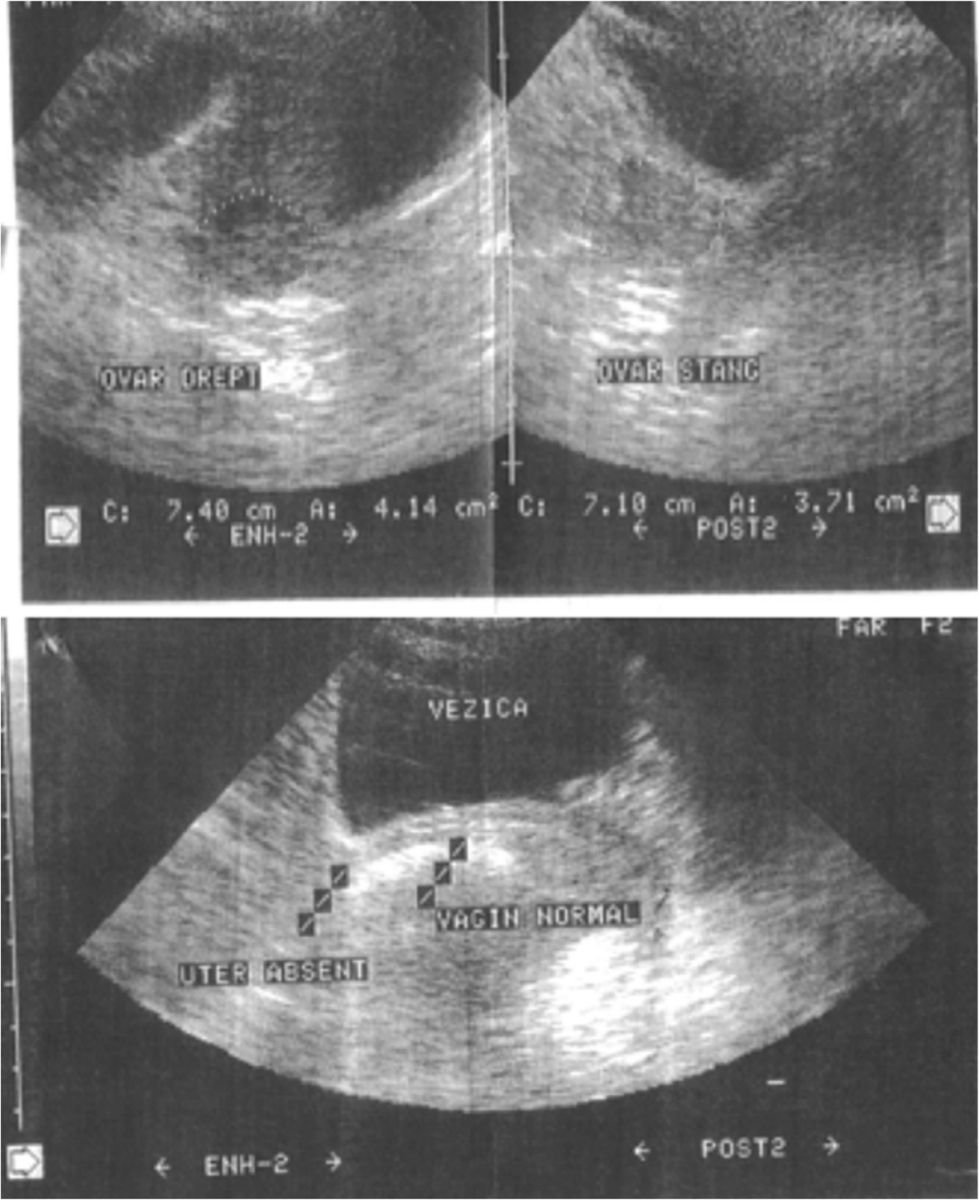
Pelvic ultrasonography images showing absence of the uterus, and the presence of both ovaries.

We obtained informed consent from our patient for diagnostic procedures and treatment, as well as consent for inclusion of her data in an anonymized scientific report. A physical examination showed that development of sexual characteristics was not entirely normal, although the development of secondary sexual characteristics was normal. Investigations included abdominal and pelvic ultrasonography and magnetic resonance imaging (MRI). Type I MRKH syndrome was suspected because of the total absence of the uterus and fallopian tubes. She had a vaginal remnant measuring 1.8cm in length. Both ovaries were normal, and no other structural abnormalities were found (Figure 1).

**Figure 2:**
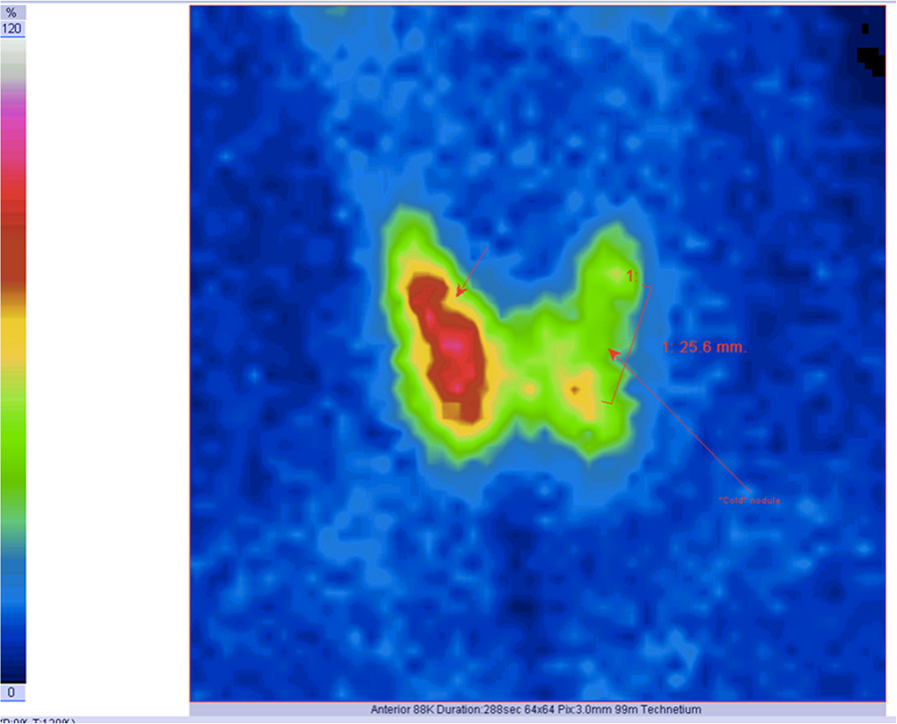
Tc-99m pertechnetate thyroid scintigraphy showing a ‘cold’ nodule in the left lobe.

Examination of the thyroid gland showed enlargement, with a mobile, 2.3cm diameter, solid nodule in the left lobe. There were multiple mobile left lateral cervical lymph nodes, with a maximum diameter of 1cm. Thyroid function testing showed normal serum levels of TSH (1.8mIU/L) and anti-thyroid peroxidase antibody (<34mIU/L). Thyroid ultrasonography showed a hypo-echoic left thyroid nodule with increased central vascularity. According to the current guidelines for evaluation of thyroid nodules [3,4], fine needle aspiration biopsy (FNAB) was indicated. The FNAB showed high cellularity in the nodule, and cytological examination showed follicular lesions that were classified as Thy3 according to the latest classification system [5]. Tc-99m pertechnetate thyroid scintigraphy (185MBq) showed a nodule in the left lobe with no metabolic activity (‘cold’ nodule) (Figure 2). The combination of clinical features, cytological examination findings, and imaging findings led to a diagnosis of nodular goiter highly suspicious for malignancy. She was referred to our Surgery Department for total thyroidectomy with selective lymph node dissection.

Pathological examination of the surgical specimen confirmed a 2.1cm diameter papillary thyroid carcinoma in the left lobe with invasion of the thyroid capsule. Three of 16 removed lymph nodes were positive for cancer. The cancer was classified as stage I (T_2_N_1a_M_x_) according to the American Joint Committee on Cancer/Union International Contre le Cancer staging system, seventh edition [6].

**Figure 3:**
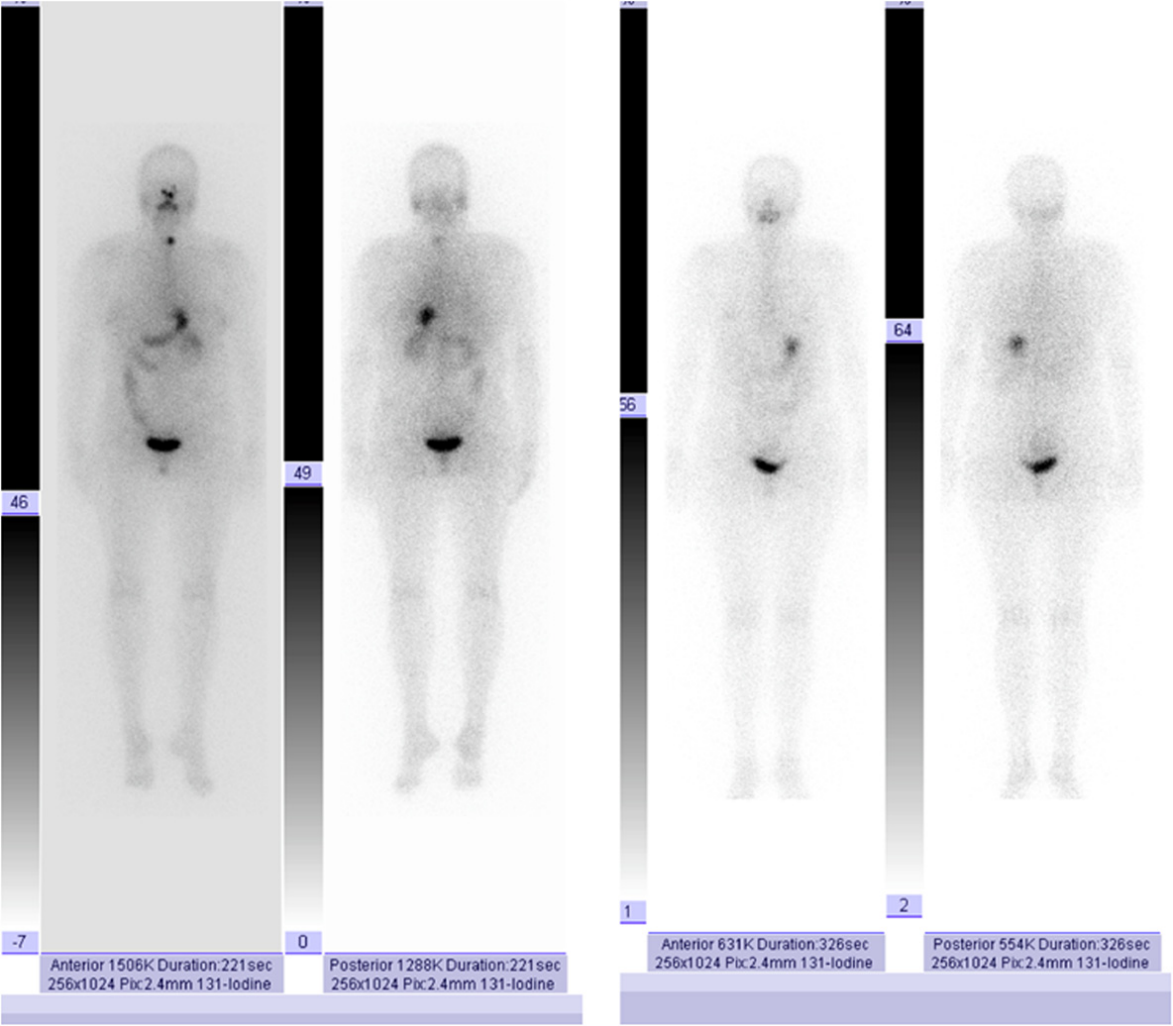
**Iodine 131 whole body scans.** (**A**) Scan three days after therapy, showing minimal residual thyroid tissue in the thyroid bed. (**B**) Scan six months after therapy demonstrating negative findings.

At four weeks after surgery, our patient underwent radioiodine therapy with a single 3.7GBq (100mCi) dose. A subsequent iodine 131 whole-body scan (I-131 WBS) showed minimal residual thyroid tissue in the thyroid bed (Figure 3A), and no evidence of lymph node or distant metastases.

Our patient started life-long suppressive hormonal replacement with l-thyroxine, 125μg daily. After six weeks of treatment, her serum TSH level was 0.083mIU/L, indicating successful suppression of the TSH.

After six months of treatment, we administered two intra-muscular injections of recombinant human TSH (rhTSH). At 48 hours after the second injection, her serum TSH level was >100mIU/L, serum thyroglobulin (Tg) level was <0.1μg/L (undetectable), and her serum anti-Tg antibody level was <10kIU/L (negative). I-131 WBS after administration of 185MBq of I-131 was negative (Figure 3B). She was considered cured of thyroid cancer, and will undergo long-term follow-up.

## Discussion

Müllerian agenesis is a congenital malformation in women characterized by developmental failure of the Müllerian ducts, resulting in absence of the uterus and fallopian tubes, and varying malformations of the upper part of the vagina. A woman with this condition is hormonally normal, and will therefore develop normal secondary sexual characteristics at puberty. Her chromosome constellation is 46,XX. The ovaries are present, and ovulation usually occurs. There are two forms of MRKH: type I results in isolated absence of the vagina and uterus, and type II also affects other parts of the body. Among those with type II, 40 percent have kidney abnormalities (15 percent of this subgroup will have only one kidney), 10 percent will have hearing problems, and 10 percent to 12 percent will have skeletal abnormalities [7]. The association of this condition with endocrine diseases is rare and a common causality has not yet been determined [8].

The cause of MRKH is unknown. Several genes have been tested to investigate a possible genetic cause, but no single factor has been identified as responsible for this condition [7,9]. Treatment consists almost exclusively of surgery, such as uterine and vaginal reconstruction, and occasionally uterine transplant.

Patients typically have a short vagina, and sexual intercourse may be difficult and painful. Women with MRKH usually discover their condition during investigation for primary amenorrhea. In this situation, most patients are evaluated for thyroid dysfunction, as some thyroid conditions may impair normal menstruation. Thyroid function and the reproductive system are connected via the hypothalamic-hypophyseal-ovarian axis.

Thyroid cancer is the most common endocrine tumor, and may be aggressive. Although the incidence of this cancer appears to have increased dramatically over the last decade [3,4], it is still considered to be rare. According to the European Surveillance of Rare Cancers Project (RARECARE), a rare cancer is defined as a tumor with an annual incidence of less than six cases per 100,000 persons [10], and all thyroid cancers fall into this category [3,4,11,12]. Thyroid cancer accounts for 0.5 percent to 1.5 percent of all childhood tumors and is the most common malignancy of the head and neck in younger people [13]. An appropriate treatment strategy can cure this disease, minimize the risk of recurrence, and result in an excellent prognosis [14].

As thyroid cancer has an incidence of six cases per 100,000 persons and MRHK has an incidence of one per 5000 women, it is emphasized that it is exceptionally rare for both to occur in the same patient. Both gynecologists and endocrinologists are encouraged to evaluate the thyroid gland in patients with amenorrhea.

## Conclusions

All women with amenorrhea should undergo complete investigation of both the genital tract and the endocrine system. The association of MRKH with thyroid cancer is currently considered to be coincidental, but further genetic research is needed to confirm the absence of a common etiology.

## Consent

Written informed consent was obtained from the patient for the publication of this case report and any accompanying images. A copy of the written consent is available for review by the Editor-in-Chief of this journal.

## Competing interests

The authors declare that they have no competing interests.

## Authors’ contributions

DP diagnosed, treated, and followed up the thyroid cancer. AP analyzed and interpreted the data, reviewed the literature, and made a major contribution to the writing of the manuscript. AI diagnosed the gynecological pathology and performed the thyroid surgery. All authors read and approved the final manuscript.
